# Digital Health Integration With Neuromodulation Therapies: The Future of Patient-Centric Innovation in Neuromodulation

**DOI:** 10.3389/fdgth.2021.618959

**Published:** 2021-05-19

**Authors:** Yagna J. Pathak, Walter Greenleaf, Leo Verhagen Metman, Pieter Kubben, Sridevi Sarma, Brian Pepin, Douglas Lautner, Scott DeBates, Alex M. Benison, Binesh Balasingh, Erika Ross

**Affiliations:** ^1^Abbott Neuromodulation, Plano, TX, United States; ^2^Department of Communication, Stanford University, Stanford, CA, United States; ^3^Department of Neurological Sciences, Rush University Medical Center, Chicago, IL, United States; ^4^Department of Neurosurgery, Maastricht University Medical Center, Maastricht, Netherlands; ^5^Department of Biomedical Engineering, Johns Hopkins University, Baltimore, MD, United States; ^6^Rune Labs, San Francisco, CA, United States

**Keywords:** digital health, neuromodulation, telemedicine, computational medicine, neuroimaging, machine learning and data analytics, wearables, digital biomarkers

## Abstract

Digital health can drive patient-centric innovation in neuromodulation by leveraging current tools to identify response predictors and digital biomarkers. Iterative technological evolution has led us to an ideal point to integrate digital health with neuromodulation. Here, we provide an overview of the digital health building-blocks, the status of advanced neuromodulation technologies, and future applications for neuromodulation with digital health integration.

## Introduction

Digital health is a field centered on improving an individual's health by combining technology with healthcare, including mobile health, wearables, telehealth and personalized medicine. In this paper, we define digital health technologies as enablers of transforming the healthcare industry into a digitalized and connected infrastructure. Basic blocks of digital health technology are mainstream, and major companies, such as Apple‡ and Google™, offer platforms for health tracking, predominantly focused on wearables and the mobile-app ecosystem. Further, several branches of medicine have adopted digital tools to improve patient care. According to a 2019 AMA report, nearly 40% of radiologists and emergency physicians use telemedicine to interact with patients and other healthcare professionals (1). Additionally, critical care units report improved patient-safety outcomes with digital health integration through the use of telehealth or computational models (2, 3).

The COVID-19 pandemic has magnified existing limitations and uncovered future opportunities to innovate methods for improved access and healthcare provider interactions. Simple tasks, such as routine visits to primary healthcare providers, are measured against exposure risk. Solutions focused on digital health to mitigate risks associated with in-person clinic visits are surging. Vulnerable populations in a viral epidemic, including the elderly and those with compromised immune systems overlap with the cohort of patients who receive neuromodulation therapies for pain and movement disorders (4, 5). Hence, the need for telemedicine and digital health solutions is amplified and there is a clear call-to-action for medical device companies to leverage existing digital tools and enable healthcare providers to manage patient wellness.

**Figure 1:**
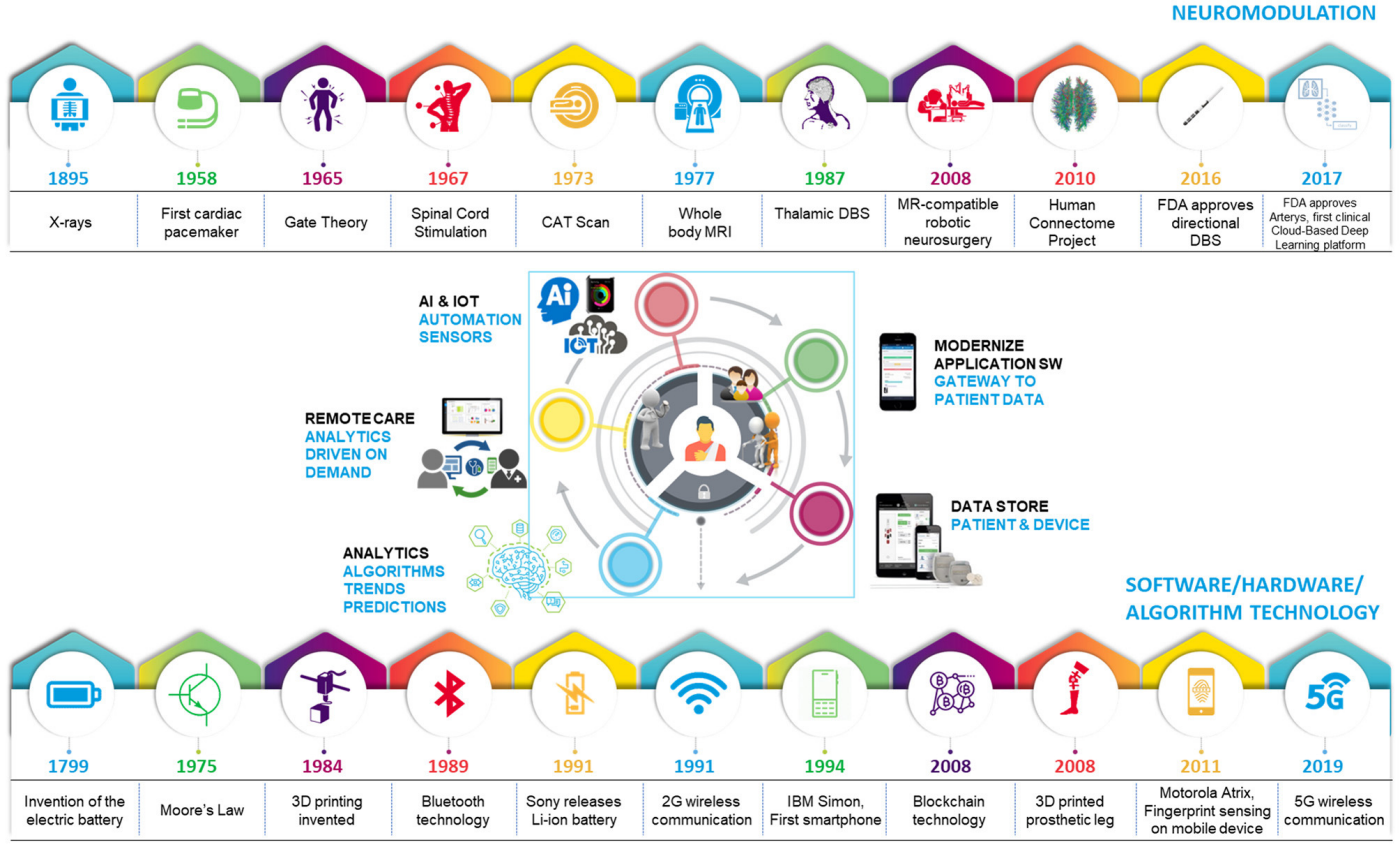
Evolution of technology leading to the age of digital healthcare. Iterative advances in technology have fueled progress in healthcare and software/hardware/algorithm spaces, leading to an optimal time for digital health integration with neuromodulation.

Despite demonstrated success in some medical specialties, adoption of digital health in neuromodulation has remained low until now. Digital health is not a panacea; effective use of digital tools requires a culture of acceptance, and guidance for active intervention that precedes data-driven predictions. These predictions also necessitate adequate understanding of diseases, and neuromodulation caters to disorders that are complex in nature. These hurdles have impeded the entry of neuromodulation into the era of big data and digital health. However, the neuromodulation field is primed to benefit from digital health integration. These tools may elevate research methodologies to holistically understand and classify health and disease states by identifying digital biomarkers with high spatial and temporal resolution and expand care access. For neurological disorders, digital biomarkers can be critical as medications have been suboptimal or contributed greatly to side-effects. Recent evidence suggests that the field is beginning to leverage tools from digital health to improve care for patients. With emerging technologies and increasing competition within the neuromodulation field, some therapy areas including spinal cord stimulation (SCS) (6) and deep brain stimulation (DBS) (7) are evolving rapidly; currently, SCS is approved for chronic, intractable pain, and DBS is approved for movement disorders [Parkinson's Disease (PD), essential tremor (ET), dystonia] and medically refractory epilepsy. Noteworthy examples of technical advancement include the application of novel waveforms (8, 9), miniaturization of neural interfaces and novel electrode geometries, and adoption of commercial mobile platforms to communicate with therapy interfaces (ex: pulse generators) and enable efficient data collection from implants (4, 5). These advances illustrate a paradigm shift toward acceptance of digital technologies and make the integration of digital health more feasible now. Advances in neuromodulation occurred in parallel to the development of digital health building-blocks, specific to software, hardware and algorithm technology, supplemented by artificial intelligence (AI). Integration of these technologies is a progressive step in bridging gaps that exist in providing truly patient-centric therapies (Figure 1).

Integrating advanced technologies from outside the neuromodulation field with state-of-the-art innovations within the field (1) facilitates personalized therapy, (2) allows physicians to evaluate continuous signals for accurate assessments, and (3) helps patients maintain their therapy without added burden. The iterative nature of technological evolution has led us to an ideal point to embrace digital health integration with neuromodulation. Here, we provide an overview of the digital health tools that are ready for integration, the status of advanced technologies in the field of neuromodulation, and applications for seamless integration of digital health technologies with neuromodulation therapies.

## Status of Digital Health Building-Blocks: Key Advances in Software, Hardware, and Algorithm Technologies

In the past decade, software and hardware technologies have advanced with far-reaching implications. Iterative innovation in software, hardware and algorithm development has resulted in key demonstrated technological successes; improvements in hardware have fueled the growth of software implementation and algorithmic integration. In turn, growing computational and connectivity needs of software solutions have driven innovation in hardware. These advances are directly related to progress in neuromodulation; improved hardware allows for finer spatial and temporal resolution, both when stimulating with complex patterns (ex: directional stimulation, burst waveforms) and sensing from neural elements, while sophisticated software enables effective aggregation of big data and creates an opportunity for algorithmic innovation in the quest for biomarkers. Further, patients' increasing familiarity with existing technology will facilitate the integration of digital health with neuromodulation therapies. The building-blocks necessary to implement effective solutions exist and are often used for alternative applications (Table 1). For example, smart phones and smart watches, that seamlessly integrate into daily life for fitness, can be leveraged for healthcare add-ons.

**Table 1:** Glossary of software, hardware, and algorithm technologies.

**Software, hardware, and algorithm technologies**	**Definition**	
5G/4G/LTE	5G is the fifth generation of wireless network connectivity for mobile telecommunication. It uses higher frequency waves compared to fourth generation (4G) options.	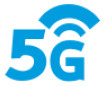
Internet of Things (IoT)	The phenomenon of allowing numerous extensions of the internet and other network connections to different sensors and devices employed in everyday use.	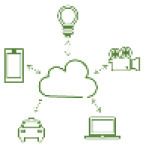
Artificial Intelligence/Machine Learning	Artificial Intelligence (AI) mimics cognitive systems by learning about its environment and problem-solving to achieve its goals, typically under the umbrella of machine leaning (ML), and natural language processing (NLP).	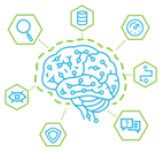
Wearables	Small computing device worn on the body. This technology includes fitness trackers, smart watches, or smart glasses.	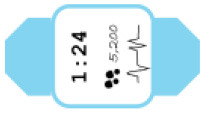
Blockchain	Blockchain is a distributed, public ledger, that records transactions and tracks assets, and of which immutability is guaranteed by a peer-to-peer network of computers instead of a centralized authority.	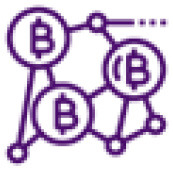
Biometrics	Unique metrics related to human characteristics, generally employed for identification, or authentication (ex: fingerprints).	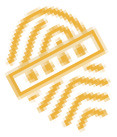
Li-ion/Li-X	Li-ion batteries are typically used in portable electronics and can be re-chargeable. During discharging phase, lithium ions move from the negative electrode to the positive electrode through an electrolyte. Due to dramatic decreases in cost and improvements in efficiency, Li-ion batteries comprise of about one-third of the battery market and since 2015, the automotive segment has been its key driver. Li-X refers to variations using unconventional anodes to improve theoretical density.	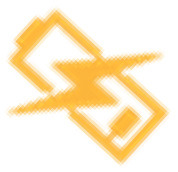
Energy Harvesters	Energy harvesting is a process in which ambient energy is converted to electrical energy. Some examples include solar farms that are aimed at harvesting photovoltaic energy or leveraging piezoelectricity with materials like quartz.	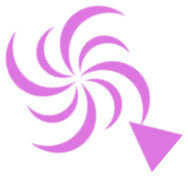
3D/4D Printing	3D printing is an additive manufacturing process where plastic, metal, ceramics, powders or living cells are deposited, or fused layer by layer to make usable solid objects from 3D model data.	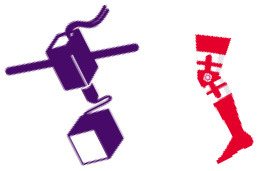
Telemedicine/Telehealth	Telemedicine is often used interchangeably to telehealth, but its scope is narrower. Telemedicine is the practice of medicine conducted remotely, including diagnosis and symptom monitoring, whereas telehealth includes non-clinical applications in addition to preventative, promotive, and curative care delivery.	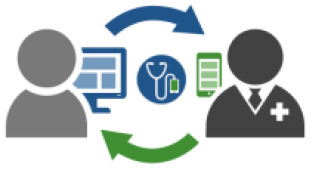
Virtual Reality	Virtual reality (VR) technologies provide an immersive experience that allow users to navigate through digital environments to carry out specific tasks. Recent studies have explored how VR can complement neuromodulation techniques.	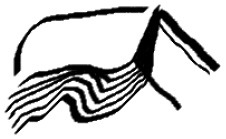

**Figure 2:**
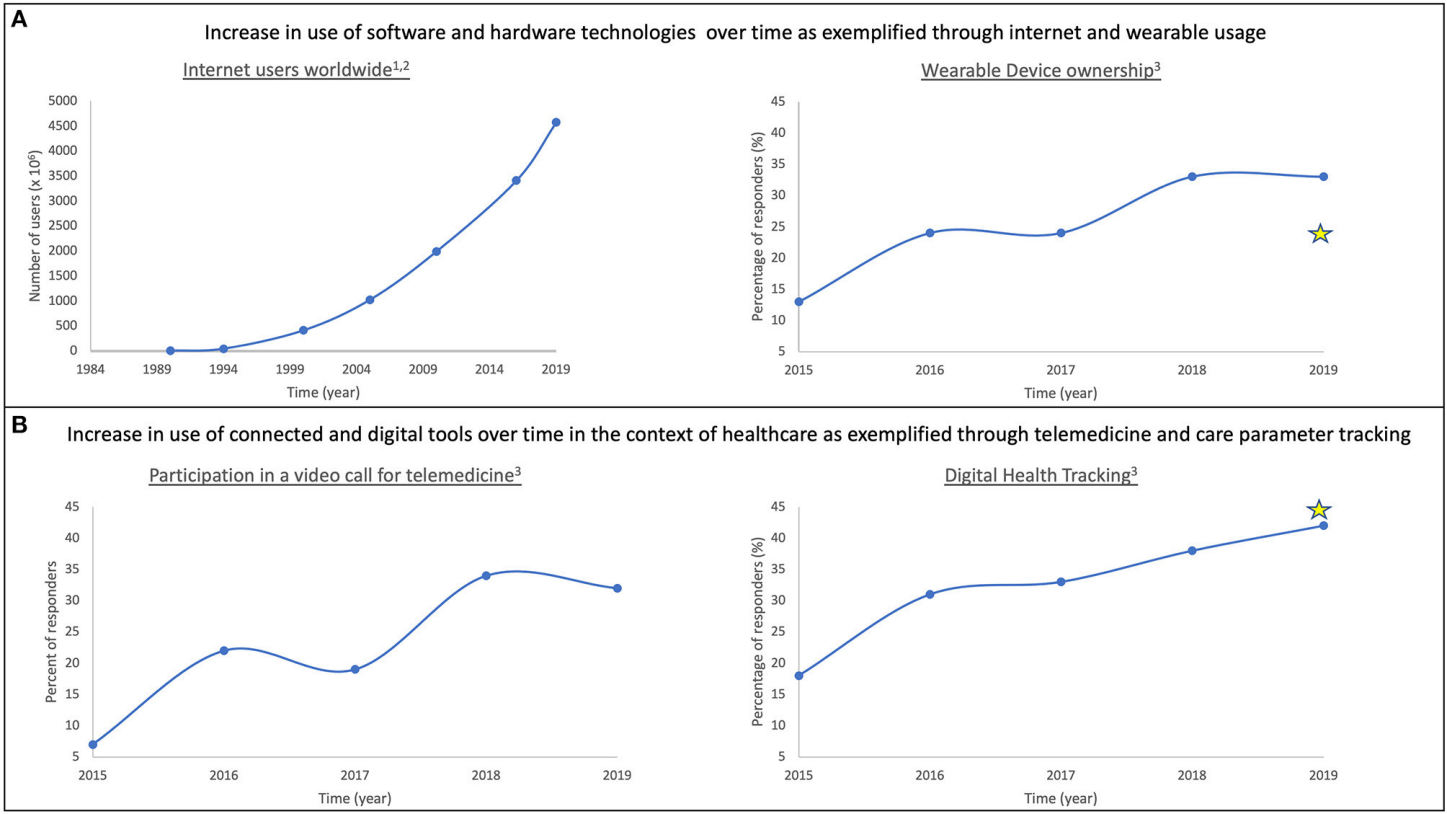
Adoption of technology over time as it becomes more ubiquitous. Prevalence of modern technology use in daily life is characterized in the context of software and hardware **(A)** and healthcare **(B)**. In both spheres, adoption of technology has increased over time. Examples included here are: internet usage (**A**: left); wearable ownership (**A**: right); participation in video-based telemedicine (**B**: left); and acceptance of digital health tracking tools (**B**: right). Stars represent responses from Abbott surveys to DBS/SCS/DRG patients. (1) https://www.visualcapitalist.com/30-year-timeline-world-wide-web/; (2) https://www.internetworldstats.com/stats.htm; (3) Rock Health Digital Health Consumer Adoption Survey (2015–2019).

In this section, we outline technological advancements that serve as stepping-stones on the path to digitalizing healthcare, due to the ubiquitous, portable and secure, real-time functional, and energy-efficient nature of these successes (Figure 2).

### Internet of Things (IoT)

Today, we rely heavily on an ecosystem of connected devices to accomplish daily tasks. From mobile apps and wearables to cloud storage, we expect constant connectivity. Internet of Things (IoT), the phenomenon of allowing numerous extensions of the internet or other networks to different sensors and devices, has radically changed computing (10). Currently, the ubiquity of wireless networks, including Wi-Fi™, 3G, and 4G/LTE, is essential to enable digital health solutions for neuromodulation that are trending toward holistic systems, connecting patients, clinicians, and caregivers.

Fifth generation (5G) of wireless transmission technology will overcome limitations of bandwidth and latency, associated with 4G/LTE, that currently impede implementation of high-quality digital health. Improved connectivity contributes to the ubiquity of digital health such that several medical devices can connect to each other and to a central cloud computing platform. 5G transmission enables the user to choose based on their requirements; network congestion may be decreased due to low latency, high bandwidth and durability, or the user may modulate data transfer speeds as a trade-off to battery life. For example, users with higher power consumption needs may opt for inexpensive hardware that allows for battery longevity, but slower data transfer speeds.

### Wearables

Other advances in connectivity technology such as Bluetooth®, coupled with better tools for data analytics, enable wearable devices to be integrated in healthcare (11). Wearable technology is important for the future of neuromodulation as it offers a non-invasive medium to collect continuous metrics in real-world scenarios that may correlate to disease state.

As noted in Figure 2A(right), wearable ownership has increased over time. Common use of wearables enables them to be integral elements of connected health-tracking systems. Specifically, popularity of devices such as Apple‡ watch and Fitbit^®^ facilitate the creation of standardized, cost-efficient and high-resolution infrastructure for resulting data. Additionally, progress in soft microfluidics and flexible electronics contributes to improved batteries and building of smart systems; smart watches, fitness trackers, and smart garments integrated with mobile technologies are ideal for continuous monitoring of activity and behavior (12, 13).

Coupled mobile applications can be expanded to include tracking of physiological parameters. To gain and retain traction in clinical practice, wearable devices must be light-weight, portable, and robustly functional when interfacing with the body. Low-powered wearable systems interfaced with mobile technology for healthcare monitoring, also known as body area networks, are emerging because of their ability for passive and wireless data transmission and storage. Continuous, real-time data acquisition facilitates personal health monitoring and provides support for clinical decision-trees that can inform physicians about patients' disease-states in a real-world setting. This defining feature is a significant puzzle piece in the growing need for personalized medicine (14, 15).

### Maintenance of Patient Records/Identification

With the emergence of wearables and inter-device connectivity, patient records will need to accommodate for increasing data, tracking patients over time. Patients who receive neuromodulation therapy begin their clinical visits long before their surgery. A longitudinal database of their condition, effects of medication and ultimately, the effect of neuromodulation, is captured through notes and imaging data in the electronic medical records (EMR). Data analytics and machine learning techniques, such as natural language processing (NLP) can be employed to extract key information from clinical notes to supplement physician decision-making (16). While EMR enable seamless tracking of clinical outcomes, security measures should be improved to better protect patient data and implement a centralized system for inter-institutional communication. With the goal of interoperability and ease-of-access, the Department of Health and Human Services (HHS) proposes new rules requiring information sharing in an accessible format by standardizing application programming interfaces (API) (17).

Improved accessibility of data needs to be coupled with proper security measures; blockchain technology and biometrics integrated with artificial intelligence (AI) are among the tools that can influence progress in this field. They increase data interoperability between multiple systems and support high throughput data analysis, contributing to the goals set by HHS (18). The immutability of the data stored in a blockchain is especially significant for digital healthcare as it strengthens privacy measures by increasing the threshold for potential breaches. However, despite the promising potential in management of medical records, advancing data storage, and accelerating clinical and biomedical research, blockchain technology is currently challenged by burdens of scalability and patient-engagement (19).

Biometrics, referring to anatomical, physiological or behavioral characteristics that are unique to individuals, contribute to technology for identification (20). Fingerprint sensing and facial recognition are already integrated as keys to unlock our devices (cellphones, laptops, etc.). Biometric measures may also serve as biomarkers to help diagnose and track medical conditions. Patients can opt to integrate these measures with EMR as real-world data and provide added context to their clinicians. Current research around facial recognition demonstrates that expressions associated with depression or pain are predictive of associated behavior and emotions (21). Lastly, biometrics may be improperly used if developed without accounting for rare use cases (ex: when an individual is unconscious) (20).

### Battery and Energy Harvesters

Batteries are the most ubiquitous technology we rely on today. Since Volta invented the voltaic pile, the field has focused on continuously improving safety, efficiency and affordability of our energy sources. Evolution in battery technology has been key to advances in neuromodulation technology, enabling complex patterns of stimulation with options for rechargeable and recharge-free therapy based on patient needs. Digital health solutions also rely on leading-edge technology for power efficiency in highly connected systems.

Currently, lithium ion (Li-ion) batteries are state-of-the-art. Practical advantages, high energy density, rechargeability, wide temperature operating window, low self-discharge, long lifetime, and improved cycling performance, make them useful in medical devices (22). Latest advances in Li-ion technology allow for wireless charging of implantable devices contributing to an overall improvement in the patient experience (23). Current versions of Li-ion batteries can improve on capacity by incorporating better anodes; choosing silicon instead of graphite, may improve capacity by 10-fold. Further, zinc-based configurations may provide safe alternatives to Li-ion technology. They are affordable, less sensitive to their environment, and display high volumetric capacity (24). Like Li-ion technology, configurations such as Zn-MnO_2_ and Zn-air, can also be used for rechargeable solutions.

Battery innovation also focuses on flexibility and miniaturization. Smart electronics embedded in communication or medical devices demand special mechanical properties allowing the devices to be rolled, bent, twisted or folded (24). Variations on Li-X, such as lithium-sulfur, and lithium-air, exhibit high theoretical density, making them crucial in the path forward toward flexible electronics (25). Also, carbon nanomaterial alternatives such as carbon nanotubes, graphene and their composites demonstrate light weight and flexibility, making them suitable for flexible battery development (26).

In terms of energy harvesting, current research is focused on sustainability and therefore, aimed at conversion of human biomechanical energy into usable electrical energy. Electromagnetic generators, piezoelectric nanogenerators and triboelectric nanogenerators are further explored for mechanical energy harvesting. Such energy harvesters provide reliable, light-weight power sources and resolve the necessity for conventional batteries currently used to power devices (27, 28). Energy harvesters also enable miniaturization of self-powered systems for use in IoTs. Though not implemented on a large-scale yet, dual functionality of energy harvesters as sensors and energy sources allow for broad applications of this type of sustainable technology (27, 29). In conjunction with active neural interfaces, these harvesters may be used to form body sensor networks, shifting the paradigm of medical device technology when further combined with IoT and AI algorithms (28).

### Hardware Miniaturization

Miniaturization technology is necessary to advance digital health innovation and integration with neuromodulation. Improving access to smaller targets, implementing edge computing electronics on powerful smartphones, and reducing the size of electronics in pulse-generators and wearables, are potential avenues to advance stimulation and sensing capabilities of current neuromodulation therapies.

Since Moore's predictions, the integrated circuit industry has focused on purpose-built and miniaturization technology. State-of-the art transistors are a few nanometers in size. Thus far, miniaturization of devices was propelled by advances in materials, fabrication, packaging and power sources (30). The future rests on emerging technologies such as flexible electronics, neural network processors (NNPs) to cope with the computational burden of AI processes with potential impact on nanotechnology, and 3D and 4D printing (28, 31).

Simple and low-cost processes associated with 3D printing enable the fabrication of customized and miniaturized devices (31). A step further, 4D printing integrates additive manufacturing and shape-morphing materials for personalized medical device solutions (32). These approaches can be employed for stimulating and sensing neural interfaces that are tailored to a patient's anatomy, comfort and overall neuromodulation treatment profile.

Flexible and soft electronics are also essential to implement tailored solutions, specifically for implantable and biocompatible medical devices (28). In addition to recording and sensing biological signals, these devices can also be used for stimulation (33, 34). Materials, like graphene, with advanced properties and biodegradable characteristics are instrumental in the integration of flexible electronics with medical device applications (35, 36). Lastly, when combined with IoT and AI, miniaturized flexible electronics serve as essential building-blocks for effective wearables, which are undoubtedly the future of digital healthcare (28, 37).

## Status of Key Technological Advances in Neuromodulation Therapy

Key factors driving technological evolution and innovation in the field of neuromodulation, thus far, are: (1) miniaturization of devices; (2) increased complexity of tasks implemented; and (3) improved understanding of neural response to stimulation (38, 39). Neuromodulation devices have decreased in size, owing to parallel technological advances in the field of integrated circuits and batteries, while the functional requirements from these devices have increased in complexity (40). Surgical requirements, aspiration for increased control over programming parameters and need for easy patient-interactions, have been fundamental drivers of progress, thus far. However, it is increasingly clear that neuromodulation therapies need to address the patients' conditions outside controlled clinical settings and as such, future digital solutions will have to focus on meeting the needs of patients that extend beyond their in-clinic evaluation while keeping the clinicians informed.

**Figure 3:**
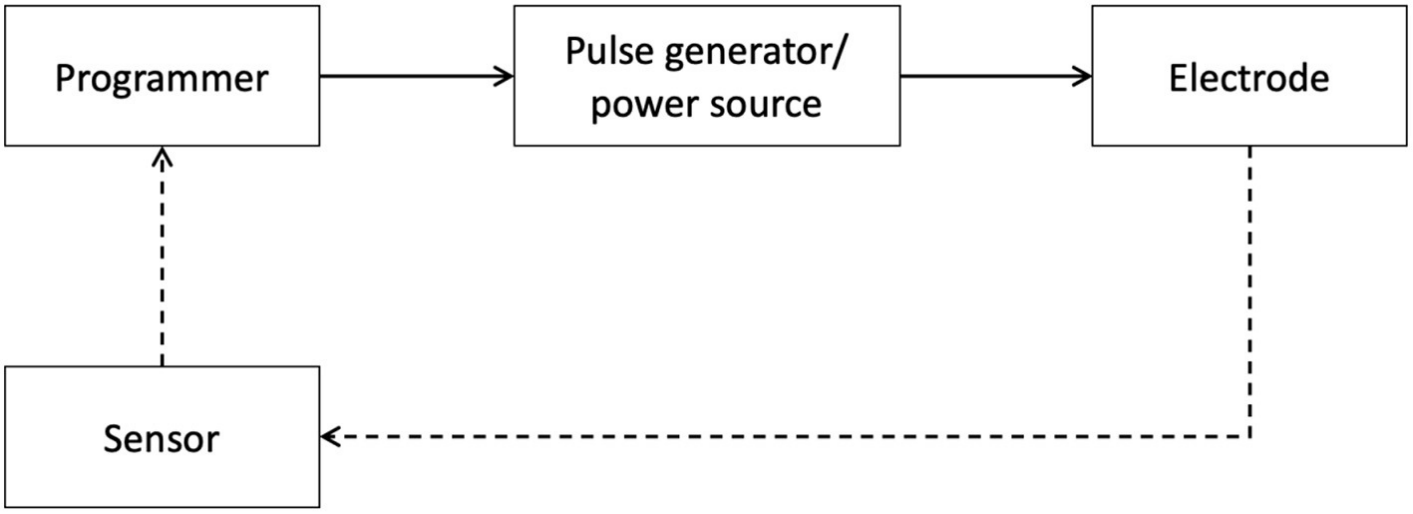
Schematic of Neuromodulation technology. A block schematic of neuromodulation technology highlights common components: the programmer, typically a clinician or a patient device, provides an interface for the user to connect with the pulse generator, which serves as a power source, and delivers specifically patterned waveforms to the neural target via the electrodes. The dashed lines represent the closed-loop paradigm now emerging in the field which integrates signals from relevant sensors to provide feedback.

Below, we expand on technological advancements in neuromodulation pertaining to diagnostics, therapy and disease maintenance. The progress made within these areas further enables the seamless integration of digital technology with neuromodulation therapy for improved patient care (Figure 3).

### Electrodes and Implantable Pulse Generator (IPG)

Electrodes and Implantable Pulse Generators (IPGs) are fundamental elements of implantable neuromodulation systems, specifically SCS and DBS. Electrodes are implanted to engage neural targets (brain, spinal cord, peripheral nerves, etc.) and are connected to an IPG, a power source that also delivers specific patterns of pulses. Typically, these devices are programmed using external devices to refine therapy in a patient-specific manner. However, conventional systems fail to account for individual variability in anatomy and physiology, leading to sub-optimal clinical outcomes. Additionally, there are continuous efforts to refine targeting and research novel stimulation patterns to optimize therapeutic effects.

With an increase in novel stimulation paradigms and sophisticated electrode geometries, programming permutations exceed what can be practically tested in-clinic. Therefore, methods to improve programming efficiency are also necessary. Current methods rely on titrating amplitude, frequency and pulse-width depending on what target is stimulated. These parameters are further refined by integrating patient-reported outcomes, evaluating symptoms and quantifying the therapeutic window (threshold between clinical benefit and elicitation of side-effects). Advanced centers employ creative approaches to optimize the stimulation field with directional electrodes (41), independent current control (42) or interleaved programming, implemented by alternating between two programs (43, 44). However, these approaches need to be refined with data-driven solutions (45).

Strides in lead development have enabled exploration of unique targets expanding the patient cohorts that may benefit from neuromodulation therapy. Advanced Neuromodulation Systems (ANS, later St. Jude Medical and now Abbott) advanced the field from the use of the simple bipolar (2-contact) interface to current quadripolar (4-contact), octopolar (8-contact), and paddle configurations.

Recently, new electrode and delivery methods were developed to target the dorsal root ganglion (DRG). Even though DRG was a focus of pain research for many years, a commercial system capable of engaging this target was not available prior to the development by Spinal Modulation Inc. in 2016. Since then, the DRG has emerged as an effective target for improvement in pain indications such as CRPS and causalgia due to its unique membrane characteristics and significance in maintaining sensory transduction (46).

In DBS, improved targeting allows us to selectively engage neural elements. Trends toward directional stimulation to improve clinical benefit and decrease potential side-effects have been fueled by innovation in electrode geometries. Directional electrodes with segmented contacts enable us to steer current away from regions of side effects and toward regions contributing to benefit, while reducing power consumption (47). White matter targets, beneficial in engaging multiple areas involved in some disorders [ex: Depression, essential tremor (ET), obsessive-compulsive disorder (OCD)], may be regions of avoidance for others (ex: PD); directional stimulation allows for selective engagement of tracts based on disease-characteristics and patients' clinical response. Additionally, multiple targets can be engaged simultaneously through co-activation. Simultaneous stimulation of pedunculopontine nucleus (PPN) and STN improved motor outcomes in a PD patient (45, 48). In one case study, authors reported that asymmetric bipolar stimulation on a directional electrode could engage STN and the ZI to alleviate symptoms for an ET patient (49). A better understanding of where to stimulate was enabled by advances in electrode technology, shifting the current focus of neuromodulation to how to stimulate potential targets, effectively and efficiently.

Smaller has always seemed better for IPGs, but computing challenges and power requirements have limited our ability to shrink these devices. Fortunately, electronic miniaturization has improved efficiency allowing for rechargeable options and comfortable primary cell alternatives for patients. Further, integration of Bluetooth^®^ low-energy (BLE) technology with IPGs is a substantial leap toward improving the user experience; it allows for interoperability of devices, while improving the speed of data transfer and widening the range of communication.

To better understand the effects of neuromodulation, neuroscience research has also focused on complex patterns of neuron firing and potential implications for information processing in the nervous system. This has led to a burst in investigations centered on novel waveforms aimed at probing the underlying neural circuitry and improving therapy. Several studies report promising results by incorporating elements of square biphasic pulses (50, 51), bursting, passive-discharge (8), spatial desynchronization (52), and pseudo-randomization (53). Improved circuit infrastructure incorporated in next generation IPGs is essential to test these patterns beyond acute settings.

### Neuroimaging and Computational Models

Though creative programming approaches are good tools to refine therapy post-operatively, imaging can be leveraged pre-operatively to improve clinical outcomes by refining targeting approaches. As mentioned, neuromodulation therapies, such as DBS, are challenging due to high inter-individual anatomical variability observed in the subthalamic nucleus (STN), a popular target to ameliorate symptoms for movement disorders. Image-guided planning for surgery provides personalized solutions that are more accurate than atlas-based targeting (54). Advances in image acquisition and computational modeling have led to better accuracy in electrode positioning (55). Further, the need to better visualize targets has driven the shift in standard-of-care for neuroimaging to stronger fields for structural magnetic resonance imaging (MRI) (1.5 tesla to 7 tesla), thereby enabling direct targeting of deep structures of the brain. Methods, such as Fast Gray Matter Acquisition T1 Inversion Recovery (FGATIR), susceptibility weighted imaging (SWI), and quantitative susceptibility mapping (QSM), are routinely incorporated in surgical planning to improve the results of MRI-guided DBS procedures (45, 56).

Integrated modalities, such as diffusion tensor imaging (DTI), and image processing pipelines further address current challenges in neuromodulation therapy. Tractography, a method to trace white matter tracts in the brain, allows for localization of dentato-rubro-thalamic tract (DRT), a target that has been efficacious for essential tremor (ET). Studies also show connectivity to be important to relieve PD symptoms; tremor improvement was associated with connectivity between primary motor cortex and superior postero-lateral part of STN while bradykinesia improvement was associated with supplementary motor area and superior postero-medial part of STN (57).

Sophisticated computational models that often integrate imaging data are valuable tools to better visualize potential effects of stimulation (58, 59). Specifically, bioelectric field models can be leveraged to plan lead placement and refine programming parameters for both, DBS and SCS (60–62). Pre-surgical models, with information about how potential stimulation will affect target neural tissue, are used to determine target coordinates. Post-surgical models can be used to determine why certain stimulation patterns are ineffective and help refine programming by examining the overlap between regions of benefit, regions contributing to side-effect and possible volumes of tissue activated (VTAs). Models can be created to be simple or complex depending on the computational power available and the required resolution of the result. Further, the quality of imaging used to generate these models defines which parameters can be tweaked (ex: tissue conductivities can be incorporated if DTI is available) (63, 64). Additionally, when testing with innovative stimulation paradigms such as novel waveforms, integrating bioelectric field models may help define safety thresholds. Overall, leveraging computational models offers an opportunity to investigate a vast parameter space and optimize neuromodulation therapy parameters in a patient-specific manner.

### Neural and Behavioral Sensing

Sensing technology used for neural and behavioral monitoring has enabled research for better biomarkers. Studies show that spike activity (65), local field potentials (LFPs) (66–68), evoked compound action potentials (ECAPs) (69) and behavioral measures such as kinematics (70, 71) are among key features that correlate with specific disease states. Intraoperative markers, such as microelectrode recordings (MER) and LFPs (72) are crucial in confirming electrode location and behavioral markers in the operating room are often measured to determine acute efficacy of neuromodulation (73).

State-of-the-art technology applied in acquisition equipment results in high quality data that may be analyzed to determine neural signal biomarkers. For invasive and non-invasive signal analysis, different materials and geometries have surged as potential topics for research to improve signal resolution, decrease presence of artifact, and stream data for storage and *post-hoc* analysis. Improved resolution of sensors and sophisticated algorithms enable identification of features previously undetectable. As technology to measure these biomarkers advance, disease states can be accurately identified allowing subsequent therapy to be optimized as well (73). Several studies have recorded synchronous activity in the beta band (13–35 Hz) from the pallidum and STN in PD patients (74, 75). Another study found that directional contacts facing posterolateral globus pallidus internus (GPi) (sensorimotor) had the largest magnitude of modulation in the 5–35 Hz range and were also chosen for clinical therapy (68). In a study focused on neurochemical signals, a wireless instantaneous neurotransmitter concentration system was employed for real-time monitoring of dopamine concentrations (76). Lastly, external sensors such as surface electromyography (sEMG) can quantify limb movement through detection of muscle contractions (45) and predict tremor onset before it appears (77).

Digital biomarkers are particularly relevant for movement disorders, psychiatric disorders and pain as they enable objective and continuous monitoring of real-world signals using digital tools (ex: mobile phones and wearables) (28). In one study, authors demonstrated that smartphone-based digital biomarkers are reliable, clinically meaningful, and sensitive by showing a correlation with the Movement Disorders Society- Unified Parkinson's Disease Rating Scale (78). In addition to movement disorders, pain studies have used indirect measures to find meaningful biomarkers. Smart phones or wearables can measure physical activity that is often negatively correlated with the level of pain (28, 79). Similarly, in a recent study, digital biomarkers, derived from passive smart-phone use, characterized neuropsychological performance (80). The ubiquity of cell-phone use and the potential for robust connectivity make these types of digital biomarkers promising to classify disease states in a time-dependent manner.

### Emerging Alternatives

The current review is focused on implantable electrical stimulation technology. However, techniques like optical stimulation and focused ultrasound are non-invasive and non-electromagnetic alternatives to DBS and SCS. Optical stimulation alludes to the use of light to trigger neurons (81). Optogenetic stimulation, a specific technique within the realm of optical stimulation, requires genetic modification of neurons to express light-sensitive ion channels that are selectively engaged with light stimulation. Despite the vast body of research around optogenetics, the technique is currently not approved for clinical use (82, 83). Other optical techniques leverage advancements in nanophotonics and in laser diode technology for infrared neural stimulation (84). Integration of optoelectronics also improves MRI compatibility; a recent vagal nerve stimulation technology (NAO-VNS) piloted the use of optical fibers and non-metal transparent components with sensing capabilities for treatment of epilepsy (85). Focused ultrasound uses non-ionizing ultrasonic waveforms to non-invasively deliver focused energy to the brain (86–88). At high intensities, the beams can be used for ablation or with lower intensities, they can be tailored for neuronal excitation or suppression (89, 90). Pulsed ultrasound technology was recently approved for Alzheimer's disease (91). While these techniques hold promise for the future, they are currently limited by complexity of implementation, non-specificity or irreversible outcomes.

## Integration of Digital Health Technologies and Future Applications With Neuromodulation

We established that the building-blocks of digital health exist, and the field of neuromodulation can benefit from digital health solutions; yet, there are practical challenges that hinder seamless integration of the two spheres. The first challenge is cultural acceptance of digital tools in neuromodulation. Though patients benefit from telehealth programs, they might be hesitant to accept telemedicine due to their expectations associated with remote vs. in-clinic visits. Further, obstacles for reimbursement, as certain tools may serve as adjunctive vs. alternative modalities to in-person evaluations, may deter clinicians when adopting these paradigms. Second, neuromodulation patients exhibit high rates of comorbidities resulting in a complex clinical plan that is refined iteratively. How can we address these comorbidities with digital tools, and can we implement an iterative approach with remote paradigms? Lastly, success of digital health integration in other fields of medicine is predicated on the ability to adequately intervene based on the data from passive monitoring. However, the complex nature of neuromodulation therapy does not allow for simple passive monitoring/active intervention approaches. Intelligent solutions are necessary to guide therapy decisions based on digitally collected data.

**Figure 4:**
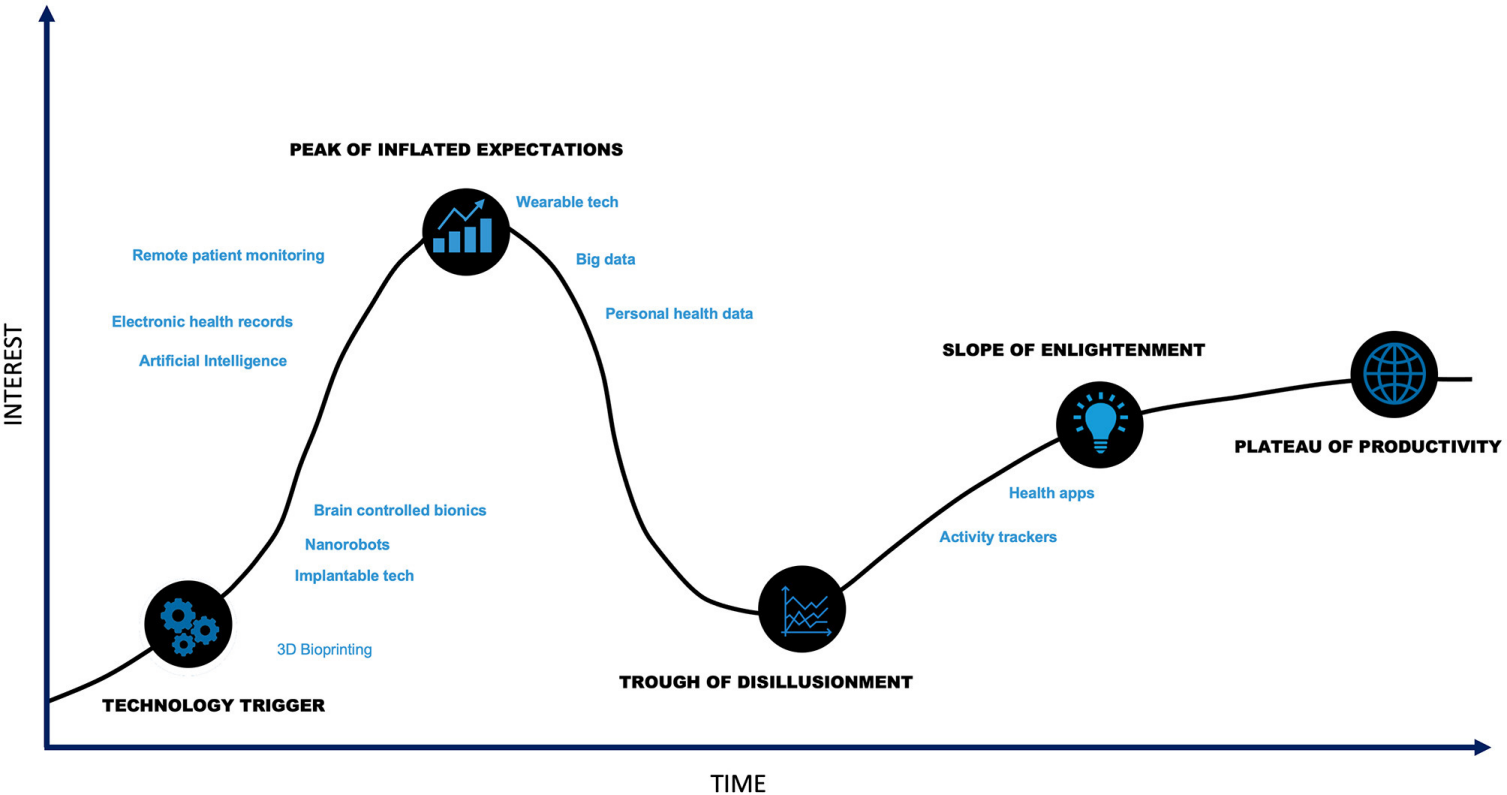
Digital Health Hype Cycle. Though digital health is at the technology trigger phase, many components of digital health technology are past the trough of disillusionment.

Innovation within digital health is trending toward addressing current challenges. Several technologies demonstrate promise in applications for diseases commonly treated with neuromodulation therapies (Figure 4). These enabling technologies and examples of their applications are outlined below.

### Telemedicine/Telehealth

In today's new normal, during a pandemic, telemedicine isn't just an option, but a necessity to avoid burdening a saturated clinical system while ensuring our own health and safety. Therefore, telemedicine is crucial for an already vulnerable population affected due to pain or movement disorders.

Recent studies demonstrated that remote programming of neuromodulation devices using telepresence is comparable to standard programming with respect to clinical outcomes (5, 92, 93). In these studies, an expert programmer is remote and instructs another clinician on the patient-end on tuning programming parameters. Future technology needs to focus on a truly remote experience which can further be leveraged for system maintenance, battery status check and device troubleshooting. When connecting remotely through these systems, patient identification and data privacy remain at the core of problems that need to be addressed. Data practices need to align with ethical principles, including respect for persons, beneficence, and justice. When data is used for healthcare related predictive analytics, transparency of potential risks should be included. Additionally, ethical concerns around the consent process need to be addressed; this includes scrutiny around open or blanket consent and sufficient detail be provided regarding primary intent for current and anticipated future use (94). Biometrics, combined with cybersecurity measures and blockchain technology, provide feasible solutions to address technical risks to data privacy. Additionally, reimbursement classification is challenging as the new way of clinical practice may not fit neatly into conventionally established categories. However, in the United States, the Centers for Medicare and Medicaid Services (CMS) began reimbursing for short virtual check-ins starting in 2019. In March 2020, CMS introduced an 1,135 waiver to expand reimbursement for telehealth interactions to further protect beneficiaries from the risk of COVID-19 (95). Additionally, payers such as Blue Cross Blue Shield (BCBS) are following suit with their versions of expanded coverage for telehealth services. Though global adoption of digital health may not be uniform, several countries are making progress toward digitization as end-users are accepting new technologies (96, 97). To adequately leverage this growing acceptance, digital health developers will need to consider creative monetization models to sustain as American solutions of reimbursement may not extend directly to European or Asia-Pacific markets. For example, in addition to publicly funded solutions, companies may consider pay-per-use or subscription-based models tailored to users. These steps are potential incentives for clinicians and patients to adopt telemedicine paradigms (98).

### Remote Patient Monitoring and Mobile Health

There is a growing need to characterize patients' disease states early because neuromodulation therapy decisions often depend on patients' constellation of symptoms. Improved image acquisition and long-term monitoring of symptoms through wearables and mobile applications are potential approaches to gather necessary data to feed into a clinical decision-tree (92, 99, 100). These measures can also be tracked after surgery to establish an objective method to quantify clinical progress. Data collected through remote monitoring can be added to EMR for holistic characterization of patients' symptoms and provide added context to care providers.

Commercial mobile platforms are an integral component of neuromodulation therapy. PainDoc^®^ (ANS), a Windows-based programming support system, pioneered the integration of familiar platforms with neuromodulation technology. Today, Apple‡ and Samsung^™^ mobile devices are integrated with proprietary applications to regulate stimulation parameters so patients can tweak their therapy depending on their symptoms and the task they need to carry out. These platforms can be expanded to improve healthcare delivery through enhanced communication, data capture, patient monitoring, education and delivery of digital interventions (101). In addition to behavioral measures that may sometimes be captured through wearables, affective symptoms can be monitored and assessed over mobile platforms.

Specifically for PD, non-motor symptoms are correlated with quality-of-life and may predict clinical outcomes (102). Therefore, there is increasing effort to digitize disease maintenance information to correlate effect of medications on symptoms and other measures. While these may start off as diaries, mobile applications have the potential to evolve into an informative tool that can help patients improve their quality-of-life.

### Virtual Reality (VR)

Virtual reality (VR) technologies may offer several clinical benefits: improving treatment efficacy, decreasing treatment time and decreasing overall cost (103). A combination of VR and telemedicine can also set the foundation for novel clinical practice in the future. Robotic surgeries are common practice, however, in combination with VR, these surgeries can be improved and potentially used for neuromodulation procedures in areas that are remote for specialists. This would increase access to a life-altering therapy for many patients.

Advances in cognitive neuro-prosthetics using VR demonstrate the capability to modulate pain perception. Several studies are investigating whether analgesic properties of SCS can be enhanced using a new system of multisensory stimulation based on VR (104).

In DBS, VR can be employed to improve placement of electrodes. VR goggles for clinicians have emerged as a potential technique to improve targeting during intra-operative physiology validation (105). Patient-specific interactive environments, as presented in VR scenarios created with patient-specific imaging (MRI/CT), may refine electrode placement for therapies where every millimeter count.

By combining immersive and realistic environments with biofeedback, VR technology can also optimize psychiatric treatments. Multipronged approaches to target differential symptom profiles are more likely to succeed (106). Hence, delivering behavioral therapy through VR environments may enhance the overall effect of neuromodulation. Several studies focused on psychiatric conditions demonstrate the effectiveness of delivering therapy over a VR platform. Specifically, behavioral exercises tailored to patients' needs can effectively engage them in their therapy and increase compliance. Further, game-like features of these tasks and realistic environments add to a heightened sense of experience (103).

### Gamification

Gamification complements neuromodulation therapy maintenance by engaging patients and by providing objective records regarding their condition (105). Several Android^™^ and iOS‡-based applications incorporate gaming elements for motor and cognitive symptoms associated with PD. Games focused on finger tapping, walking and speaking are used to track patients' motor symptoms (107). In addition, gamification applications also enable tracking of mental health and cognitive symptoms to improve memory, attention and problem solving (108). Recently, the Food and Drug Administration (FDA) approved the first game-based digital therapeutic to address attention-deficit and hyperactivity disorder (ADHD) in children. This further illustrates the shifting paradigm and cultural acceptance of digital solutions (109).

Existing commercial VR gaming systems, such as Nintendo Wii™ and X-Box Kinect™, can be leveraged for neurorehabilitation interventions to enhance gait and balance for patients with movement disorders (110). Several platforms are already set up for exergaming, where users are required to move in response to gamified on-screen tasks. Lastly, immersive systems provide a realistic environment that can be integrated in game-form for symptom tracking (111).

The goal of gamification is to decrease the burden of therapy maintenance while providing opportunities for rehabilitation. As this field evolves, a combination of machine learning (ML) and AI tools will enable applications to suggest personalized task-levels. This patient-centric approach may increase engagement and contribute to improved clinical outcomes.

### Computational Medicine

The purpose of any healthcare paradigm should ultimately be to improve patients' lives. With the technologies outlined above, frequent consultations and real-world data collection become feasible. However, the field still needs to agree on what to do with data and subsequent predictions and how to individualize interventions while accounting for comorbidities.

Improving methods for diagnoses and treatments based on insights from computational models is referred to as computational medicine (112). One computational medicine approach is to leverage data analytics and control theory models to achieve a desirable response from the system (113). Certain comorbidities may also serve as potential predictors of symptoms. For example, pain patients frequently suffer from sleep disorders. Early adoption of wearables that provide sleep measures may be useful in predicting symptoms. After receiving SCS, these measures can be monitored continuously to objectively determine the clinical response. This data can be used to create a simple, phenomenological model with the control objective to match responses observed in a healthy system, where non-noxious stimuli do not typically illicit pain. This model will eventually provide programming guidance based on patient-specific data.

## Conclusion

The future of neuromodulation is intertwined with digital health. Advanced technologies from other fields have found a promising place in healthcare and its increased use enables a seamless integration of these tools with neuromodulation therapies. Initial goals of this multimodal, digital health approach are to (1) facilitate personalized therapy, (2) allow physicians to evaluate continuous signals for accurate assessments, and (3) help patients maintain therapeutic effects without additional burden. In addition, data-driven techniques implemented through this approach will fuel innovation, improve disease understanding and lower costs while increasing access to life-altering neuromodulation therapy for affected patients. Digital health integration is a gateway for an exciting and innovative future of neuromodulation.

## Author Contributions

All authors listed have made a substantial, direct and intellectual contribution to the work, and approved it for publication.

## Conflict of Interest

YP, DL, SD, AB, BB, and ER are employees of Abbott Labs. BP is an employee of Rune Labs. WG and SS serve on the Scientific Advisory Board for Abbott Neuromodulation. The remaining authors declare that the research was conducted in the absence of any commercial or financial relationships that could be construed as a potential conflict of interest.
